# Farm Prevalence of Bovine Brucellosis, Farmer Awareness, and Local Practices in Small- and Medium-Scale Cattle Farms in a Tropical Region of Ecuador

**DOI:** 10.1155/2023/6242561

**Published:** 2023-08-26

**Authors:** R. L. Vinueza, B. Durand, F. Ortega, F. Salas, A. Ferreira Vicente, L. Freddi, C. Ponsart, G. Zanella

**Affiliations:** ^1^University Paris-Est, Anses, Laboratory for Animal Health, Epidemiology Unit, Maisons-Alfort 94700, France; ^2^Universidad San Francisco de Quito, Escuela de Medicina Veterinaria, Cumbayá, Quito 150157, Ecuador; ^3^Universidad San Francisco de Quito, Escuela de Salud Pública, Cumbayá, Quito 150157, Ecuador; ^4^Universidad San Francisco de Quito, Instituto de Medicina Social & Desafíos globales, Cumbayá, Quito 150157, Ecuador; ^5^University Paris-Est, Anses, Laboratory for Animal Health, Bacterial Zoonoses Unit, National and OIE (WOAH) Animal Brucellosis Reference Laboratory, Maisons-Alfort 94700, France

## Abstract

A cross-sectional study was conducted on bovine brucellosis in a sample of 173 medium/small-scale cattle farmers in a tropical region of Ecuador. A total of 173 milk tank samples were collected and analyzed through an indirect ELISA. A survey was also applied to each farm to collect information on herd management, evaluate the level of knowledge about the disease and estimate the risk that bovine brucellosis on those farms could represent for public health. The apparent prevalence among farms was 11.5% (95% CI: 6.7%–16.2%). The medium farms had a prevalence significantly higher (23.8%, 95% CI: 10.9%–36.6%, *p* < 0.0001) than the small farms (7.6%, 95% CI: 4.5%–9%). Two multivariable analysis were conducted to identify risk factors associated with bovine brucellosis or reproductive disorders. Medium farms had 3.7 more odds to be infected than the small farms (OR: 3.7, 95% CI: 1.39–9.84, *p* = 0.008). Incineration/burial of abortion material was identified as a protective factor (OR: 0.4, 95% CI: 0.14–0.98, *p* = 0.04). Farm size and brucellosis were identified as risk factors for the occurrence of reproductive disorders. Only 25% of the farmers were aware of bovine brucellosis. Unpasteurized curdled milk was regularly consumed and marketed on 112 farms of which 14 were positive for bovine brucellosis. Highly at-risk practices, such as manipulation of aborted fetuses were also reported. These results indicate that there is a need to develop public health education programs targeted at medium/small-scale farmers in Ecuador.

## 1. Introduction

Brucellosis is a widespread zoonosis, mainly affecting developing countries where it causes economic loss and threatens public health and food security [1]. It is caused by bacteria of the genus *Brucella* that can affect many wild and domestic animals. Although there are several potentially dangerous *Brucella* species for humans, most infections are caused by *B. abortus* and *B. melitensis* [2]. Cattle are the primary source of infection in humans [3].

*B. abortus*, the bovine brucellosis pathogen, affects not only cattle but also humans. In cattle, transmission occurs through contact with excretions, secretions, and reproductive discharges of infected animals, ingestion of contaminated feed and water, natural breeding, and in some cases, through artificial insemination with contaminated material [4]. The most common manifestations of the disease in cattle are reproductive disorders, including abortion during the last third of gestation, infertility and stillbirths [5]. In addition, the milk is contaminated for life in positive animals [6] and production is compromised by the prolongation of calving intervals [7]. The economic impact for the farmer is substantial, due to losses from abortions, extension of days-open or without pregnancy, commercial restrictions on both infected animals and their products, and costs associated with efforts to eliminate the disease on the farm, among others [8, 9]. In Latin America, losses due to the presence of the disease would reach US$600 million per year [10].

As a zoonosis, bovine brucellosis poses a high risk for people who have close contact with cattle such as farmers, animal caretakers, veterinarians, or personnel working in slaughterhouses [11–13]. In humans, the infection can occur directly and indirectly. The direct form includes contact with aerosols or uterine secretions or placentas from diseased animals by handling abortions or laboratory samples [14]. The indirect form can occur by consuming contaminated unpasteurized milk or cheese [15]. Human infection manifests with fever, chills, sweating, general malaise, anorexia, headache, joint pain, back pain, fatigue, and muscle pain [12]. Sufferers may eventually develop severe forms of the disease, including endocarditis, arthritis, orchitis, and neurological disorders [16], requiring prolonged periods of antibiotic treatment and long convalescence [9].

In Ecuador, where cattle are the main livestock species, bovine brucellosis is widespread [17], except for the island province of Galapagos, which may be considered as free [18]. In a study conducted in semiextensive dairy and mixed farms in the main milk-producing provinces in Ecuador a between-herd prevalence of 45% and a within-herd prevalence from 10% to 100% were found [19]. Economic loss due to the disease has been estimated to US$5.5 million per year [20].

Brucellosis in humans has been reported by the Ministry of Public Health of Ecuador in several provinces [21], most cases being directly related to the presence of the disease in the bovine population [22, 23]. Little is known about the role played by small- or middle-cattle farms in the maintenance and dissemination of brucellosis in Ecuador. Certain social aspects as well as infrastructure and livestock management in rural areas can be risk factors for the spread of brucellosis within and between the farms [24].

In 2008, the Animal Health Agency of Ecuador launched the National Bovine Brucellosis Control Program [17]. This strategy included vaccination with strain 19 in calves from 3 to 6 months, epidemiological surveillance using the Rose Bengal test followed by the competitive ELISA to confirm, elimination of positive animals, strengthening of diagnostic laboratories, and even the sensitization of farmers, promotion and dissemination of the program. The program did not provide for any compensatory measures covering the removal of positive animals, or economic incentives for small-scale farmers. Therefore, the program implementation costs would be borne by the farmers. Given the noncompulsory nature of the program implementation and the high costs of the disease diagnosis, the medium/small-scale farmers have not get involved [25, 26]. Commercial farm owners are the only who have joined the program and they receive a compensation per liter of milk supplied to milk processing plants. In 2016, a manual of procedures for the control of bovine brucellosis was issued by the Ecuadorian Veterinary Services in 2016 that incorporates the possibility of using the strain RB51 vaccine, leaving the farmer free to choose the type of vaccine [7].

This study was conducted in dairy and dual purpose (meat and milk production) medium/small-scale farmers, in a tropical region of Ecuador (Quinindé). The objectives were: (i) to estimate the bovine brucellosis prevalence at the farm level, (ii) to identify potential risk factors associated with brucellosis positive farms or reproductive disorders, (iii) to assess the level of knowledge of the disease among farmers, and (iv) to analyze the risk that the occurrence of brucellosis in these farms could represent for public health.

## 2. Material and Methods

### 2.1. Study Area

Quinindé is one of the seven cantons (intermediate administrative unit) in the Esmeraldas province (Ecuador is divided into 24 provinces) located in the coastal region at 190 meters above the sea level. Its temperature oscillates around 25°C, with a relative humidity above 80%. Around half of the farms and cattle population of Esmeraldas province are located in the Quinindé (∼4,200 farms and 135,000 cattle). Most of the farms are mixed farms (cultivation of crops combined with cattle farming) of dual purpose raising zebu (*Bos indicus*) and its crosses with European breeds.

### 2.2. Study Design

The study was conducted between March and November 2017.

For the sampling size estimation, a between-farm prevalence of 11%, a confidence level of 95% and a precision of 5% were considered. A sample size of at least 135 cattle farms was obtained using the WIN EPI 2.0 program [27]. We looked for increasing that number to at least 170 farms to conduct the identification of risk factors. The farm census information from the foot and mouth disease information system of Ecuador was used for the farm selection. Of 4,200 small-cattle farms present in Quinindé, 1,772 were retained for the random sampling because they were small or medium farms and had a record of dairy or dual-purpose activity.

### 2.3. Milk Sampling and Laboratory Analysis

Milk samples of 100 ml were taken in each farm from the milk collection tanks, and kept in sterile jars. The samples were collected at milking in the morning in their raw state, making sure not to contaminate the contents. The samples were transported in refrigerated thermal boxes, maintaining the cold chain, to the laboratories of the Universidad San Francisco de Quito, where they were stored at −20°C, until the date of shipment to the National and WOAH Animal Brucellosis Reference Laboratory from Anses located in Maisons-Alfort, France, where they were analyzed.

An antibody test was performed using and indirect ELISA Brucellosis Milk Ab Test from IDEXX. The short incubation protocol was performed following the procedures described by the manufacturer.

### 2.4. Survey Design

A questionnaire including multiple choice and open questions was designed to collect information on the following topics: farm characteristics, veterinary assistance, herd management, milk and dairy products trade, milk and cheese consumption, animal health management, and farmer brucellosis awareness.

The questionnaire was first validated within a group of 10 farmers from a neighboring canton. Following this validation, some questions were adjusted to facilitate the understanding of the farmers of the area.

The information was collected with the support of final year veterinary students and local health technicians, who received prior training.

### 2.5. Statistical Analysis

Between-farm apparent prevalence was calculated based on the ELISA results.

Two multivariable analyses were conducted using a logistic regression to identify risk factors linked to farm brucellosis status according to milk seropositivity on the one hand, and, on the other hand, farmer reporting of reproductive disorders. The bovine brucellosis serological status of the farm (i.e., the ELISA result obtained from the milk collected in the farm) and the occurrence of reproductive disorders were the binary outcomes. The farm size was categorized into two categories: small farms (<50 cattle) and medium farms (≥50 cattle).

Sixteen variables were considered for the identification of risk factor linked to farm status. Multicollinearity between these variables was checked to ensure a variance inflation factor (VIF) <2. The procedure to select the variables for the final multivariable model is described in [28]. In brief, univariable associations were tested and the variables with *p* < 0.2 were retained to be included in the multivariable analysis. The least significant variables were then removed using a backward and forward stepwise procedure. Only variables with *p* < 0.05 were retained in the final model.

Concerning the occurrence of reproductive disorders, bovine brucellosis status of the farm and farm size were considered as risk factors and tested directly in a multivariable analysis.

Statistical analysis were performed using the RStudio software version 1.4.1717 and the epiR, car, and MASS packages [29]. The georeferenced maps were obtained in shape file format and scale 1 : 50,000 from the Geoportal of the Ecuadorian Military Geographic Institute (http://www.geoportaligm.gob.ec/), and processed in Qgis 3.6.0 Noosa (QGIS.org, 2020).

## 3. Results

### 3.1. Sample Description

The farm sample was composed of 131 small farms (1–50 cattle) and 42 farms of medium size (51–200 cattle). One-third of the surveyed farms reported cattle raising as the main economic activity (35/131 small farms and 34/42 medium farms, Fisher's exact test: *p* < 0.0001); all the others had also agriculture activities. The average surface was 22 hectares for small farms, and 33 hectares for medium farms.

All farms managed cattle extensively, with cultivated pastures. In most cases, pastures were subdivided for rotational grazing. In times of drought, some farmers rented pastures (37/131 small and 12/42 medium; Fisher's exact test: no significant difference (NS)) and some of those with irrigation rented their pastures and received animals from other farms.

All herds were of mixed type. The proportion of dairy cows was higher in the medium farms than in the small ones (22% in medium farms and 6% in small farms; Fisher's exact test: *p* < 0001). Almost all farms had other animal species: horses/mules (147/173), pigs (72/173), and dogs (160/173). Sheep or goats were not reported on any of the farms.

None of the farms visited had potable water. A minority of farms had non-potable piped water (40/131 small farms and 22/42 medium farms; Fisher's exact test: *p* = 0.03), while the others had access to sources such as wells and reservoirs or streams shared with neighbors.

Most farmers replaced their cattle with cattle from their own farm (101/131 small farms and 25/42 medium farms; Fisher's exact test: *p* = 0.03), and the others bought cattle at markets or from neighbors. About 70% of farmers sold their cattle to traders that came to the farm and 29% at markets or to neighbors.

Natural breeding was implemented in 169 farms (127/131 small farms and 42/42 medium farms; Fisher's exact test: NS), most of the times using their owned breeding bull. Artificial insemination was implemented in three small and four medium farms. Calving occurred in dedicated pens in 17/173 farms (5/131 smalls farms and 10/42 medium farms; Fisher's exact test: *p* < 0.0001).

Only a small group of farmers (8/131 small-scale farmers and 9/42 medium-scale farmers; Fisher's exact test: *p* = 0.007), belonged to a cattle association. One-third of the farms (33/131 small farms and 26/42 medium farms; Fisher's exact test: *p* < 0.0001) had veterinary assistance. This technical veterinary assistance was provided mainly by state organizations (mainly for the small farms: 25/33) or veterinary companies (mainly for the medium farms: 11/26).

### 3.2. Farm Apparent Prevalence Estimation

**Figure 1:**
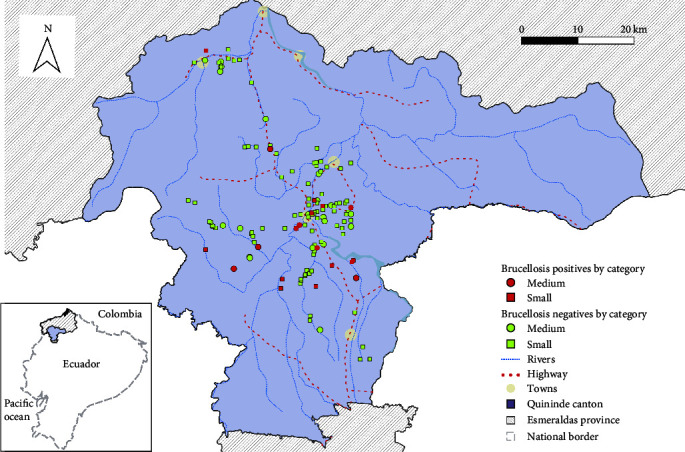
Brucellosis-positive and -negative cattle farms according to serological result in milk tank from 131 cattle farms sampled in Quinindé canton, province of Esmeraldas, Ecuador.

The overall apparent between-farm prevalence was 11.5% (95% IC: 6.7%–16.2%). In the group of medium farms, the between-farm prevalence (23.8% and 95% IC: 10.9%–36.6%) was significantly higher (Fisher's exact test: *p* = 0.02) than in the group of small farms (7.6% and 95% IC: 4.5%–9%). The spatial distribution of farms according to their ELISA result is shown in Figure 1.

### 3.3. Risk Factor Analysis for Bovine Brucellosis Status of Farms

The 16 potential risk factors for bovine brucellosis included in the univariable analysis are described in Table 1, according to the farm brucellosis status. No collinearity was detected among those variables.

In the multivariable analysis, farm size and incineration/burial of abortion material were identified as significantly associated with farm brucellosis status (Table 2). Medium farms had 3.7 more odds to be infected than the small farms (OR: 3.7, 95% CI: 1.39–9.84, *p* = 0.008). The incineration or burial of the abortion material was a protective factor (OR: 0.4, 95% CI: 0.14–0.98, *p* = 0.04). When not incinerated or buried the abortion material was in general left in the pastures or could also be given to the farm dogs.

### 3.4. Risk Factor Analysis for the Occurrence of Reproductive Disorders in Farms

Reproductive disorders were reported in 23 medium farms (55%) and 32 small farms (24%). Farm size and brucellosis-positive status were found to be associated with their occurrence (Table 3). The odds of reproductive disorders was 2.9 times higher in medium-sized farms than in small farms (OR: 2.9, 95% CI: 1.07–8.06, *p* = 0.002). Reproductive disorders were reported in 12 farms positive for *Brucella* (60%) and eight farms negative for *Brucella* (40%). The farms positive for brucellosis had 3.3 higher odds of having reproductive disorders than the negative farms (OR: 3.3, 95% CI: 1.55–-6.89, *p* = 0.008).

### 3.5. Awareness of Farmers about Brucellosis

Forty-four of the 173 farmers surveyed (i.e., 25%) mentioned having heard about the the disease (29/131 small-scale farmers and 15/42 medium-scale farmers, Fisher's exact test: NS). However, only 15 of these 44 farmers were aware of the brucellosis transmission route between animals, the proportion being significantly lower in small-scale farmers (5/29) than in medium-scale farmers (10/15) (Fisher's exact test: *p* = 0.002). The proportion of farmers who knew that the disease was zoonotic among those 44 farmers was significantly higher in medium-scale farmers (10/15) than in small-scale farmers (7/29) (Fisher's exact test: *p* = 0.009).

Vaccination against brucellosis was performed in four farms (1/131 small-scale farms and 3/42 medium-scale farms) (Fisher's exact test: *p* = 0.044). The strain 19 vaccine was applied in one small-scale farm and in three medium-scale farms. Strain RB51 vaccine was also used in one medium-scale farm.

Ninety-eight of 173 farmers (57%) showed interest in participating in a control and prevention plan and 21/173 (12%) considered the process too complicated. Seven farmers were not interested in participating in a control and prevention plan because they assumed it would be too expensive.

### 3.6. Public Health Risk

Fresh milk was marketed directly or through intermediaries on 36/131 small farms and 19/42 medium farms (Fisher's exact test: *p* = 0.04). Of these farms, seven were positive for bovine brucellosis. Raw milk was occasionally consumed in 10 small farms (two were positive for bovine brucellosis) and seven medium farms (two were positive for bovine brucellosis) (Fisher's exact test: NS). Curdled unpasteurized milk was regularly consumed on 112 farms (87/131 small farms and 25/42 medium farms) of which 14 were positive for brucellosis (4/87 small farms and 10/25 medium farms; Fisher's exact test: *p* < 0.0001).

Cheese was produced on 153/173 farms (117/131 small farms and 36/42 medium farms), of which 18 were bovine brucellosis positive (8/117 small farms and 10/36 medium farms; Fisher's exact test: *p* = 0.001). It was sent to markets by 83 farms (57/131 small farms and 26/42 medium farms), of which 17 were positive farms (8/57 small farms and 9/26 medium farms; Fisher's exact test NS), or consumed on 156 farms (119 small farms and 37 medium farms), of which 18 were positive (8/119 small and 10/37 medium; Fisher's exact test: *p* = 0.002). The cheese was produced using traditional methods, and none of the farmers reported cheese pasteurization.

Four small-scale farmers reported that they consumed the aborted fetuses.

Six medium-scale farmers reported having had a family member diagnosed with brucellosis in the last 5 years (none among the small-scale farmers, Fisher's exact test: *p* = 0.002).

## 4. Discussion

Bovine brucellosis in Ecuador has not been sufficiently studied. As in other Latin American countries, bovine brucellosis continues to be underdiagnosed and underreported [3, 30], so its prevalence is probably underestimated. On the other hand, most studies on bovine brucellosis in Ecuador have been carried out in industrial and semi-industrial milk production systems, which constitute barely 30% of the country's total cattle farms. No studies on bovine brucellosis had been conducted previously in small and medium-sized farms that represent 92% of the total productive units registered in the country [31]. These farms are considered family farms of dual-purpose (agriculture and livestock raising) with little technology [32]. The aims of our study were to provide information on brucellosis prevalence, risk factors, level of knowledge of the disease among farmers, and identify risk practices in small and medium farms in a tropical region of Ecuador.

We found an overall farm bovine brucellosis prevalence of 11.5%. This value is below the one reported (45%) in a study carried out in commercial dairy and mixed farms in the Ecuadorian central provinces [19] or in a study conducted in commercial mixed cattle farms (22%) in Manabí province [23]. In neighboring countries, such as Colombia, where surveys have been conducted in productive farms of the main dairy regions, prevalence values between 22% and 43% were reported at the herd level depending on the region [8, 33].

We also found that the bovine brucellosis prevalence in medium-sized farms (23.8%) was significantly higher than in small farms (7.6%). In addition, the size of the farm was identified as a risk factor for the presence of brucellosis. It has also been the case in other studies leading the authors to point out that the largest farms are more exposed to the disease due to more cattle movements from or to markets or to the introduction of new animals for growth or fattening [34–36]. In Ecuador, even though in the manual of procedures for the control of bovine brucellosis it is established that moved cattle need a health certificate showing that they are free from brucellosis [7], in practice, most farmers do not require this document for transactions. Therefore, the probability of introduction of the disease into a bovine brucellosis free farm is higher for farms purchasing more animals [37]. The medium-size farms included in our study did not have the technology level of big commercial farms, but they were more often involved in livestock trading than the small farms. A similar situation occurred in the largest herds of dairy goats in Ecuador where higher prevalence values of brucellosis were found [38].

Burying/incinerating fetal remains was identified as a protective factor in our study. The survival of *Brucella* in the environment has been reported in several studies [39, 40]. Specific characteristics allow the pathogen to survive in moist soil at room temperature for up to 66 days, in manure for up to 80 days, in leather stained with cow dung for 21 days, and even in water at 8°C and a pH of 6.5 for more than 57 days [39]. Some authors claim that *Brucella spp*. can resist in the soil with a relative humidity of 40% for several months at 4–8°C, and 144 days at 20°C [41]. Other authors have pointed out that an average temperature of 18°C and a high-relative humidity may favor the persistence and spread of the disease [42]. Those humidity and temperature conditions are fully met in the tropical zone of Ecuador. In the current study, it was observed that it is a common practice for some farmers to leave the remains of abortions in the pasture or to allow their access to dogs. This practice has also been reported in other studies as a risk factor [43]. Moreover, dogs have been shown to act as reservoirs and vehicles for the spread of *Brucella abortus* on livestock farms [44], reason why some authors suggest that dogs could also be sentinels for the detection of this disease [45]. Well-managed breeding systems (including isolation of aborting animals) can help reduce the prevalence of infection within the herd [46] and proper disposal of aborted materials and highly hygienic procedures constitute crucial steps in any successful *Brucella* control program [34].

Some risk factors included in our study were not significant but should be considered for the proper control of the disease, among them; calving in the open field, the fate of the cow that aborts, the management of manure, the origin of the animals, the use of ditch water from other farms, the lack of vaccination and immunity of livestock, the type of reproduction, and the biosecurity measures applied at the farms [47].

Brucellosis and farm size were linked to the occurrence of abortions in our study. In areas where brucellosis is endemic, abortions and retained placentas may raise suspicion about the presence of the disease in the first place, mainly when abortion occurs in the last third of pregnancy [48, 49]. However, it should be taken into account that not all abortions can be attributed to brucellosis, and not all animals infected with brucellosis will necessarily abort. Abortions in Ecuador could also be attributed to other infectious agents such as infectious bovine rhinotracheitis, bovine viral diarrhea, or leptospirosis [50, 51].

Different strategies have been undertaken in Ecuador to control the brucellosis. Some have been successfully implemented in the commercial farms but they have not reached the small-producer sector. It could be due to the lack of official technical assistance. It has been reported that less than half of the medium farms in Ecuador have access to the technical assistance [52]. In this study, it was found that only a third of farms had access to technical assistance and that in most cases it was provided by veterinary commercial companies whose interests may not include the official sanitary measures guidelines.

In our study, only 11% of farmers knew how bovine brucellosis was transmitted to cattle. Although some farmers had heard of the disease, most did not understand its transmission mechanism or that it was a zoonosis. This brucellosis unawareness would not be limited to this region. Indeed, in a study carried out in the Ecuadorian Andean region, it was pointed out that indigenous communities were unaware of the disease [53]. In another study including 500 farmers of the province of Manabí in Ecuador, only 30% knew about brucellosis, and 7.6% about the measures to reduce its transmission [54]. The lack of knowledge of the disease among people involved in bovine production (farmers, slaughterhouse workers, cattle, and milk dealers) has also been reported in other regions of the world. In a meta-analysis including 22 countries in Asia, Oceania, Africa, North, South, and Central America it was found that only 35% of the interviewees knew about the disease, and 36% that it was a zoonosis [55]. Brucellosis awareness could also be improved through the participation to farmers' associations. In our study, although there was a significant difference between the two groups of farmers, few of them belonged to a cattle association. In contrast, half of the farmers showed interest in participating in a prevention program but some found the process complicated or too expensive. Therefore, different aspects may be playing a role in the lack of interest of small- and medium-scale farmers from the study region to participate in the national brucellosis control program that include low-brucellosis awareness, absence of economic incentives, and lack of guidance/support from the authorities.

In the Ecuadorian coastal region, the consumption of raw curdled milk and fresh cheese made on the farm is a typical practice. The commercialization of fresh milk through intermediaries was significantly higher in the group of medium-scale farmers than in the small ones. The lower participation of small-scale farmers in milk trade could be explained by their low-production yields and little bargaining power with the intermediaries [56]. The more intense exchange by medium-scale farmers also means that milk from farms where brucellosis prevalence is higher could be reaching places farther from the production centers. The significant lower participation of small-scale farmers in cattle associations could be affecting their access to the market since the associations can obtain better prices for their products.

Considering that a third of the farmers interviewed in this study marketed curdled milk and about 90% fresh cheese, including those whose farms were positive for brucellosis, this practice constitutes a danger not only for the farmers but also for those who consume the products in urban centers. In Colombia, the presence of *Brucella* has been confirmed in samples of fresh artisanal cheese [57]. In an outbreak of *Brucella melitensis* in humans reported in Spain [58], 81 human cases had a strong association with the consumption of fresh cheese made by an artisan in an establishment without control. In Ecuador, the presence of *Brucella* in products such as cheese has not been studied. Some of the interviewed farmers also reported the consumption of aborted fetuses. The consumption of bovine placenta or aborted fetuses is an alimentary tradition in some regions of Ecuador and has been linked with the occurrence of human cases of brucellosis not through the consumption of the food item, which is cooked, but due to its manipulation [59]. Although human brucellosis is considered primarily an occupational zoonosis among farmers, veterinarians and slaughterhouse workers, the consumption and handling of contaminated food or remains of abortions, and poor hygiene, can significantly increase the risk of the disease in the general population [60]. In our study, six medium-scale farmers mentioned having had a relative affected by brucellosis in the last five years. Considering that bovine brucellosis prevalence was significantly higher in this category of farms, people living in these farms would be more exposed to brucellosis infection. Finally, human brucellosis underdiagnoses cannot be excluded in the region. Some authors have pointed out that in many cases, the symptoms of brucellosis in humans can be mild or infrequent, so the diagnosis is not even considered [61]. Additionally, in the tropical countries such as Ecuador, some symptoms of brucellosis in humans can be confused with malaria and typhoid fever [55].

In conclusion, bovine brucellosis circulates in small- and medium-sized farms in a tropical zone of Ecuador with a higher prevalence in the medium-sized farms. The lack of bovine brucellosis awareness as well as some local practices that may influence the spread of the disease in cattle and humans were identified. For these reasons, to control the disease, it is necessary to make small- and medium-scale farmers aware of brucellosis through information and health education campaigns. Warnings about the consumption of raw milk/cheese or the manipulation of aborted fetuses should be included in these campaigns. This first approach in the control of the disease is necessary to make acceptable other control measures, including vaccination.

## Figures and Tables

**Table 1:** Descriptive statistics of potential risk factors included in the univariable analysis according to farm brucellosis serological result in milk tank from 131 cattle farms sampled in Quinindé canton, province of Esmeraldas, Ecuador.

Features	Potential risk factor	Category	Positive/total (%)
Cattle replacement	Farm size ^*∗*^	Small	10/131 (8)
		Medium	10/42 (24)
	From neighboring farms	Yes	3/29 (10)
		No	17/144 (12)
	From the same farm ^*∗*^	Yes	12/126 (10)
		No	8/47 (17)
	Purchased at fairs ^*∗*^	Yes	6/24 (25)
		No	14/149 (9)

Calving	Having calving pens	Yes	1/17 (6)
		No	19/156 (12)

Reproduction	Natural breeding	Yes	19/169 (11)
		No	1/2 (50)
	Insemination	Yes	0/7 (0)
		No	20/166 (12)

Abortion management	Incineration/burial of abortion material ^*∗*^	Yes	9/115 (8)
		No	11/58 (19)
	Keeps the aborting cows	Yes	17/140 (12)
		No	3/33 (9)

Manure management	Leave manure in the barn	Yes	13/119 (11)
		No	7/54 (13)
	Use manure as fertilizer	Yes	4/44 (9)
		No	16/129 (12)
	Manure runs off into a stream	Yes	2/10 (20)
		No	18/163 (11)

Share of pastures and water sources	Pastures shared with cattle from other farms	Yes	15/146 (10)
		No	5/27 (19)
	Water source shared with other farms ^*∗*^	Yes	12/75 (16)
		No	8/98 (8)

Others	Share cattle utensils with other farms	Yes	1/3 (33)
		No	19/170 (11)
	Cattle vaccination against brucellosis ^*∗∗*^	Yes	0/1 (0)
		No	20/172 (12)

^*∗*^Variables found significant at *p* < 0.2 in the univariable analysis,  ^*∗∗*^Strain RB51/strain 19.

**Table 2:** Results of the multivariable logistic regression on the farm brucellosis status according to serological result in milk tank from 131 cattle farms sampled in Quinindé canton, province of Esmeraldas, Ecuador.

Risk factors	Categories	Odds ratio	95% CI	*P*-value
Farm size	Medium	3.7	1.39–9.84	0.008
	Small	Reference		

Incineration/burial of abortion material	Yes	0.4	0.14–0.98	0.04
	No	Reference		

**Table 3:** Results of the multivariable logistic regression model on the occurrence of reproductive disorders in 131 cattle farms sampled in Quinindé canton, province of Esmeraldas, Ecuador.

Risk factors	Categories	Odds ratio	95% CI	*P*-value
Farm size	Medium	2.9	1.07–8.06	0.002
	Small	Reference		

Brucellosis positive farms	Positive	3.3	1.55–6.89	0.04
	Negative	Reference		

## Data Availability

The data that support the findings of this study are available from the corresponding author upon reasonable request.
